# Effect of gamma radiation on storability and functional properties of sorghum grains (*Sorghum bicolor* L.)

**DOI:** 10.1002/fsn3.752

**Published:** 2018-08-30

**Authors:** Manahel Mohammed Ahmed, Ismat G. Abdalla, A. M. Salih, Amro B. Hassan

**Affiliations:** ^1^ Department of Food Science and Technology Faculty of Agriculture University of Khartoum Khartoum Sudan; ^2^ Institute of chemistry and Nuclear Physics Sudanese Atomic Energy Commission (SAEC) Khartoum Sudan; ^3^ Environment and Natural Resource and Desertification Research Institute (ENDRI) National Center for Research Khartoum Sudan

**Keywords:** functional properties, gamma irradiation, protein digestibility, sorghum, storability

## Abstract

This study was aimed to evaluate the effect of gamma irradiation at dose levels of 0.5, 1.0, 2.0, 3.0, 4.0, and 5.0 kGy on fungal growth, free fatty acids, in vitro protein digestibility (IVPD), protein solubility, and functional properties of sorghum grains. Results indicated that radiation process enhanced the storability properties. It eliminates the fungal incidence particularly at the higher doses 4.0 and 5.0 kGy, and significantly (*p* < 0.05) reduced the content of the free fatty acids to the level 3.4–3.2 mg/g. Moreover, a significant increase in IVPD and soluble protein was noticed in irradiated grains as compared to non‐irradiated sample and this increased with the increase in dose; however, maximum IVPD (17.6%) and protein solubility (11.7%) were observed in sorghum grains irradiated at 2.0 kGy. On the other hand, a significant reduction in emulsion capacity was observed after radiation of grains, however, the emulsion activity and stability were stable up to 1.0 kGy. The foaming properties of the radiated sample show no significant change particular at low doses up to 2.0 kGy when they are compared to untreated samples. It can be concluded that low doses of gamma irradiation might improve the storability and quality characteristics of sorghum grains and can be used as an effective alternative postharvest method for preserving and extending the shelf life of sorghum and its products.

## 1 INTRODUCTION

In most of the semi‐arid regions of Africa and Asia, including Sudan, sorghum (*Sorghum bicolor* L.) considered as an important staple food. People in these countries, particularly in Sudan, used sorghum as a source of food, fermented beverages, malt, feed, fiber, and biofuel (Mutegi et al., 2010; Muyanja, Kikafunda, Narvhus, Helgetun, & Langsrud, 2003). Like other types of grains, sorghum is stored in many different ways and for different periods of time and susceptible to infest by different types of store pests during storage. Commonly, chemicals are used for disinfestation purposes; however, extensive application of these chemicals used for controlling store pests negatively impacts on the environment and store products (Cherry, Abalo, & Hell, 2005). Thus, several physical alternative methods have been suggested for postharvest pest control. Among these alternative methods, low doses of gamma radiation method has advantages in inhibiting the microbial growth in food (Iqbal, 2016; Bhatti, Iqbal, Anwar, Shanid, & Shahid, 2013; Mbarki, Sadok, & Barkallah, 2008), remove the toxicity from textile effluents (Iqbal & Nisar, 2015), enhancing the germination and seedling growth of seeds (Hussain et al., 2017), and enhanced its shelf life (Brewer, 2009). Furthermore, gamma radiation up to 6 kGy maintained the oil characteristics of almond (Bhatti et al., 2013).

Moreover, most of the previous studies stated that the nutritional and functional characteristics of grains are improved after radiation treatment particularly at low doses (Mukisa et al., 2012; Hassan et al., 2013; Osman et al., 2014; Mahmoud et al., 2016). However, the safe recommended gamma doses for elimination pest as well as to maintain the quality of the products should not exceed 10 kGy (Mbarki et al., 2008). Furthermore, former studies stated that gamma radiation enhances the functional properties of plant materials. According to Hassana, Mahmouda, Elmamounb, Adiamoc, and Mohamed Ahmed (2018), irradiation of sesame seeds at dose level up to 2.0 kGy did not denature the protein and enhanced its functional properties. Improving the functional properties of proteins is desirable for the utilization of any new protein material since they affect the protein behavior during the food processing, which governs the quality properties of the final product (Kinsella, 1976).

It has been stated that the interest in sorghum as a gluten‐free cereal is increased to substitute the gluten‐rich cereals in the diet of people suffering from celiac disease (Elkhalifa, Schiffler, & Bernhardt, 2005). Therefore, improving the functional properties of sorghum proteins is desirable for the purpose of supplementation or replacement of the toxic protein sources. Low doses of gamma radiation expected to improve the functional properties of sorghum seeds, since irradiation can cause changes in protein functionality as fragmentation and aggregation can occur when protein is irradiated. Therefore, in this study, the radiation treatment of sorghum experiments was designed for a maximum dose 5.0 kGy to investigate the effect of gamma irradiation at various doses on storability properties, nutritive value and functional properties of sorghum grains.

## 2 MATERIALS AND METHODS

### 2.1 Sample collection and preparation

Sorghum sample (Fetarita cultivar) was obtained from the Strategic Reserves Department at the Agricultural Bank of Sudan. Grains were freed from the foreign materials, broken seeds and impurities, and then stored at 4°C during the analysis. All reagents used in this study were of reagent grade.

### 2.2 Radiation treatments

Sorghum grains were sealed in plastic bags and exposure to gamma rays at dose levels of 0.5, 1.0, 2.0, 3.0, 4.0, and 5.0 kGy with dose rate 1.3 kGy/hr at Kaila irradiation processing unit, Sudanese Atomic Energy Corporation (SAEC) using an experimental cobalt‐ 60 gamma source (Nordion gamma cell 220—Excell). To ensure uniform dose delivery and minimize the variations in radiation received by the samples both sides of the sample bags were exposed to gamma rays (double side irradiation). Three dosimeters (Gafchromic HD‐810 film, International Specialty Products, NJ, USA; FAO/IAEA/USDA 2003) were included in each batch of the seeds and read after irradiation with Radiachromics reader (Far West Technology Inc., CA, USA) to measure the dose received by the batches. Triplicate samples of the sorghum grains were irradiated, and all treatments were repeated three times. Nonirradiated seeds served as control.

### 2.3 Fungal growth determination

The parentage of the fungal growth of raw and radiated samples was determined according to standard methods (AOAC, 1995) after platted on double strength Sabaroud Dextrose Agar and incubated at 25 ± 2°C for 5 days.

### 2.4 Free fatty acids content determination

Free fatty acids (FFA) content of sorghum grains were measured according to Aibara, Ismail, Yamashita, and Ohta (1986). FFA content was calculated as mg KOH required to neutralize them in one‐gram grain on dry matter basis.

### 2.5 Crude protein, in vitro protein digestibility and protein solubility determination

The crude protein of the samples was determined using micro‐Kjeldahl method (AOAC, 1995). The in vitro protein digestibility (IVPD) was estimated according to Maliwal (1983). Determination of the protein solubility in sorghum samples was performed according to the method described by Hagenmaier (1972).

### 2.6 Emulsification properties

The emulsion capacity (EC) of the irradiated and raw sorghum flour was estimated according to the method described by Beuchat (1977). The EC was expressed as the millimeters of oil emulsified or held per gram of sample. The emulsification activity (EA) was determined according to Yasumatsu et al. (1972). The EA was expressed as percent of the emulsified layer from the total volume.

(1) EA(%)=Height of emulsified layer/Height of total volume×100

Emulsion stability (ES) was determined by a procedure described by Yasumatsu et al. (1972), by re‐centrifugation followed by heating at 80ºC for 30 minutes and then cooled to 15ºC. The ES was calculated as the percent of the total volume remaining emulsified after heating.

(2) $$\begin{array}{cc} \text{ES}\;(\%)= & \text{Height of emulsified layer after heating}/\\ & \text{Height of total volume}\times 100 \end{array}$$

### 2.7 Foaming properties

Foaming properties, foaming capacity (FC), and foam stability (FS), were determined according to Coffman and Gracia (1977). About 0.7 g of the sample was whipped with 100 ml distilled water for 5 min in lab rotary blender and then poured into a 250 ml measuring cylinder and the foam volume was recorded. The FC was calculated according to the following equation.

(3) $$\begin{array}{cc} \text{FC}\;(\%)= & (\text{volume after whipping}-\text{volume before whipping})/\\ & \text{volume before whipping}\times 100 \end{array}$$

The foam stability (FS) was calculated by measuring the decrease in volume of the foam in the measuring cylinder after stood for 30 min.

(4) FS(%)=foam volume after30min/initial foam volume×100

### 2.8 Statistical analysis

The data were statistically evaluated by the one‐way analysis of variance procedure (ANOVA). The least significant difference test (LSD) was applied to compare mean values. All analyses were performed in triplicate (*n* = 3). The level of significance used was 95%.

## 3 RESULTS AND DISCUSSION

### 3.1 Effect of gamma irradiation on storability properties of sorghum grains

**Figure 1:**
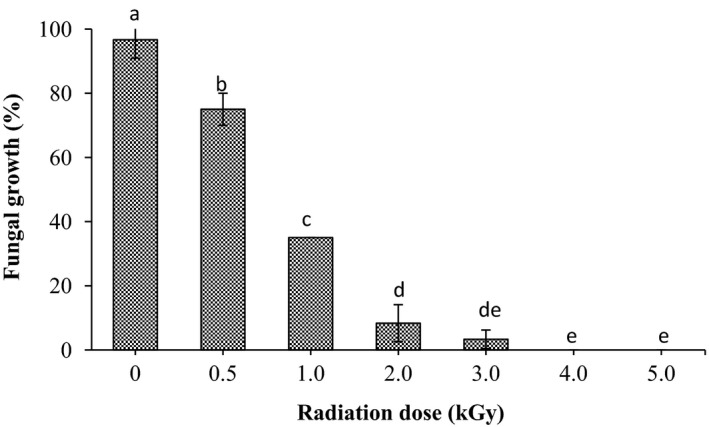
Effect of gamma radiation on fungal growth of sorghum. Values are means (±*SD*) of triplicate samples. Values followed by the same letter are not significantly different (*p* < 0.05) as assessed by LSD

Figure 1 describes the fungal growth in sorghum grains before and after radiation. The fungi growth in sorghum grains was found to be 100% before radiation treatment. It was clearly observed that radiation treatment significantly (*p* < 0.05) reduced the fungal incidence in sorghum grains. As radiation dose was increased the inhibition of fungal growth increase. It was found to be 75, 35, 8.3, 3.3 % for dose levels 0.5, 1.0, 2.0, and 3.0 kGy, while no fungal growth was observed at high doses 4.0 and 5.0 kGy, respectively.

**Figure 2:**
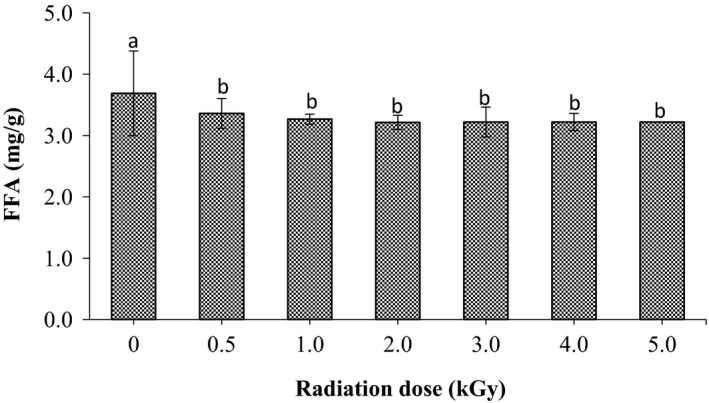
Effect of gamma radiation treatment on free fatty acids (FFA) of sorghum. Values are means (±*SD*) of triplicate samples. Values followed by the same letter are not significantly different (*p* < 0.05) as assessed by LSD

Figure 2 shows the effect of gamma radiation at different dose levels on the free fatty acids (FFA) content in mg/g of sorghum grains. As shown in the figure radiation process significantly (*p* < 0.05) reduced the content of FFA in sorghum compared to untreated one. The FFA content of sorghum flour was found to be 3.69, 3.36, 3.27, 3.21, 3.22, and 3.22, 3.22 mg/g at dose levels of 0.0, 0.5, 1.0, 2.0, 3.0, 4.0, and 5.0 kGy, respectively.

From the obtained results, it is noticed that gamma radiation of sorghum grains enhanced its storability properties in term of fungal growth and formation of the free fatty acid. The inhibition of the fungal growth in sorghum grains after radiation might be due to the destruction of their DNA since they are highly sensitive to gamma radiation (Refai, Aziz, El‐Far, & Hassan,^1996^; McNamara, Black, Beresford, & Parekh, 2003). Moreover, our results agree with the findings described by several studies. In a study conducted by Aziz and Moussa (2004), radiation dose up to 3.5 kGy was required to eliminate the fungi growth in many fruit and vegetables. Mahmoud et al. (2016) concluded that gamma radiation of millet grains up to 2.0 kGy caused a significant reduction in it's, both, fungal incidence and free fatty acids content. On the other hand, Iqbal (2016) encouraged the application of gamma irradiation as a protective method against the fungal growth and aflatoxin production. On the other hand, the reduction of FFA content after radiation treatment might be due to the reduction of lipase in treated grains, which result in dropping the FFA formation. Pankaj, Kudachikar, and Sourav (2013) that ionizing radiation causes a reduction in the lipase activity in wheat germ have reported it. Thus such reduction of the FFA formation in radiated samples may be enhanced its storability quality since they are response for the rancidity in stored grains.

### 3.2 Effect of gamma irradiation on in vitro protein digestibility and protein solubility of sorghum grains

**Figure 3:**
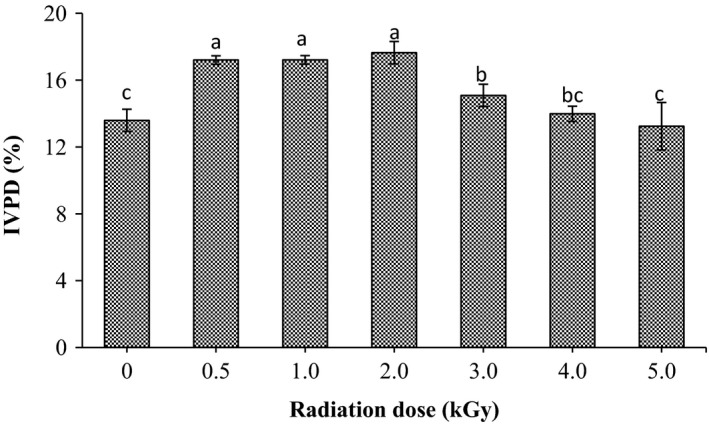
Effect of gamma radiation treatment on in vitro protein digestibility (%) of sorghum. Values are means (±*SD*) of triplicate samples. Values followed by the same letter are not significantly different (*p* < 0.05) as assessed by LSD

The effect of gamma radiation on the in vitro protein digestibility (IVPD) of sorghum is shown in Figure 3. As observed in the figure, the IVPD of sorghum grains is significantly (*p* < 0.05) affected by gamma radiation. It was found to be 13.6% prior the radiation, progressive increase in radiation dose up to 2 kGy concurrently increased the IVPD of the grains to 17.7%. However, at doses >2 kGy the IVPD of grains decreased significantly (*p* < 0.05) to 15.1%, 13.98% and 13.24% for radiation doses 3.0, 4.0, and 5.0 kGy, respectively.

**Figure 4:**
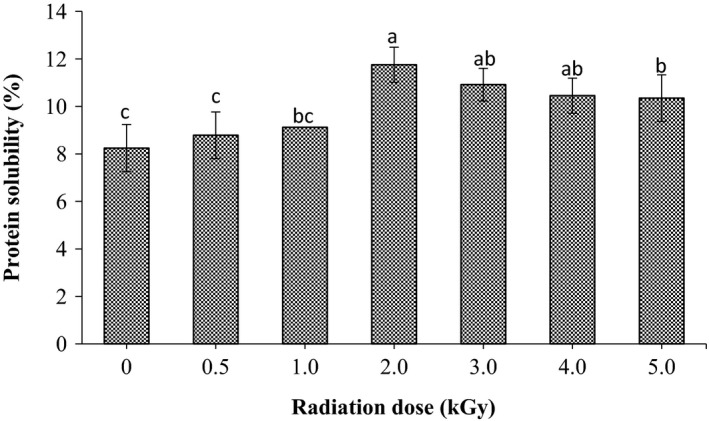
Effect of gamma radiation treatment on protein solubility (%) of sorghum. Values are means (±*SD*) of triplicate samples. Values followed by the same letter are not significantly different (*p* < 0.05) as assessed by LSD

As shown in Figure 4, the protein solubility of untreated sorghum was found to be 8.24%. It was clearly observed that radiation has a significant impact on protein solubility particularly at doses higher than 2.0 kGy. At the dose of 2.0 kGy higher value in protein solubility was recorded. Conversely, it was gradually declined upon using higher doses of gamma radiation 3.0, 4.0, and 5.0 kGy; however, it was still significantly higher than the non‐irradiated grains.

For both digestible and soluble protein, it was found that gamma radiation significantly increased their values as radiation dose increased up to 2.0 kGy then gradually declined upon using higher doses. Similar results had been reported by Mostafa (1987) who stated that protein digestibility of groundnut increased with the increase in radiation dose; however, it was gradually declined at doses up to 3.50 kGy. Moreover, Bamidele and Akanbi (2013) and Bhat, Sridhar, and Seena (2008) stated that low dose of gamma irradiation significant increase the in‐vitro protein digestibility of pigeon pea flour and velvet beans seed, respectively. Increasing of IVPD after radiation might be due to the reduction in the antinutrients particularly tannin content of grains (Osman et al., 2014; Mahmoud et al., 2016). Similarly, Rehman and Shah (2001) reported that gamma radiation lower antinutritional factors such as phytates and tannins, thereby creating a large space within the matrix of flour that enhance susceptibility to enzyme attack and consequently increase protein digestibility. On the other hand, the enhancement of the IVPD and protein solubility of radiated sorghum samples at low doses up to 2.0 kGy might be due to the fact that gamma radiation cause changes in the structure of protein molecules by disrupting of the di‐sulfide and hydrogen bonds (Koppelman et al., 2005). These changes will lead to improving the proteolytic activity and hydrolysis of the peptide bonds of the proteins. Additionally, the high protein solubility in irradiated grains at 2.0 kGy dose could be attributed the presence of lesser exposure of hydrophobic residues and repulsion of unfolded protein molecules in irradiated samples at this dose. Further decrease in protein solubility at higher dose levels could be due to progressive denaturation of sesame proteins with increasing irradiation dose. Similar observations were reported in cowpea proteins irradiated at different doses (Abu, Muller, Duodu, & Minnaar, 2006). Conversely, the reduction in protein digestibility and solubility of sorghum grain at higher doses, more than 2.0 kGy, might be due to denaturation of the protein caused by exposure of hydrophobic groups and aggregation of unfolded protein molecules (Afify, Rashed, Mahmoud, & El‐Beltagi, 2011).

### 3.3 Effect of gamma irradiation emulsifying properties of sorghum grains

**Figure 5:**
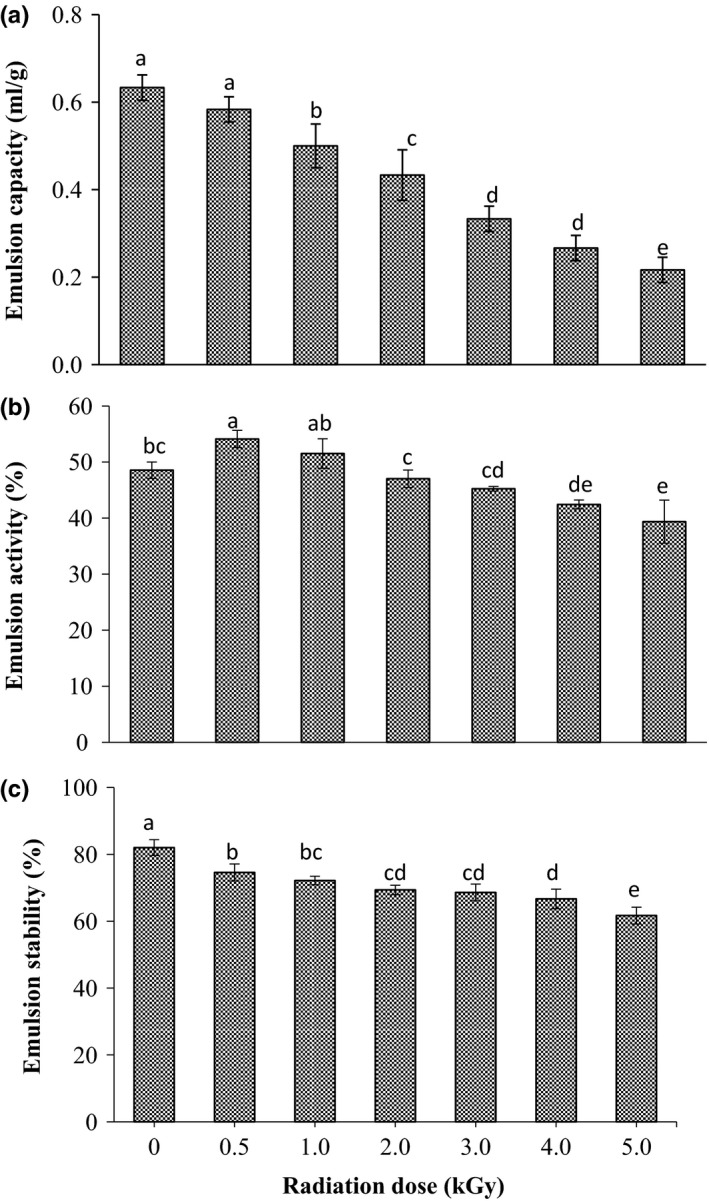
Effect of gamma irradiation on emulsion capacity (a) emulsion activity (b) and emulsion stability (c) of sorghum. Values are means (±*SD*) of triplicate samples. Values followed by the same letter are not significantly different (*p* < 0.05) as assessed by LSD

Figure 5a–c describe the emulsion capacity (EC), emulsion activity (EA) and emulsion stability (ES) of sorghum grains before and after radiation treatment. Before radiation the emulsion capacity of sorghum grains was found to be 0.63 mg/ml (Figure 5a). From the figure, it was clearly observed that gamma radiation causes a significant (*p* < 0.05) reduction on the EC of sorghum grains, it was decreased gradually as the dose was increased. Regarding the data presented in Figure 5b, the emulsion activity of sorghum flour concurrently (*p* < 0.05) increased as the radiation dose increased up to 1.0 kGy. However, at doses higher than 1.0 kGy the EA of the flour significantly decreased to the lower level less than the control sample. On the other hand, the emulsion stability was shown a dose‐dependent decreased. As the radiation dose was increased, the emulsion stability significantly (*p* < 0.05) decreased (Figure 5c).

The obtained results showed that the emulsifying properties were significantly (*p* < 0.05) change after radiation. Reduction of emulsion capacity and emulsion stability might be due to the denaturation and aggregation of sorghum protein, particularly at high radiation doses. Several researches stated that gamma radiation has a variable impact on the emulsifying properties of the agricultural crops. These differences could be due to the genetic variation, protein profile and gamma doses. Enujiugha, Olotu, and Malomo (2012) and Abu et al. (2006) found that gamma radiation significantly reduced the emulsion capacity of African oil bean seeds and cowpea seed, respectively. In contrast, radiation process enhanced the emulsion capacity of soybean seeds and sun flower seeds (Pednekar, Das, Rajalakshmi, & Sharma, 2010; Malik & Saini, 2017). Denature of protein which occurred due to radiation process might be the main factor of degradation of the emulsion properties of sorghum grains particularly at doses higher than 1.0 kGy.

### 3.4 Effect of gamma irradiation foaming properties of sorghum grains

**Figure 6:**
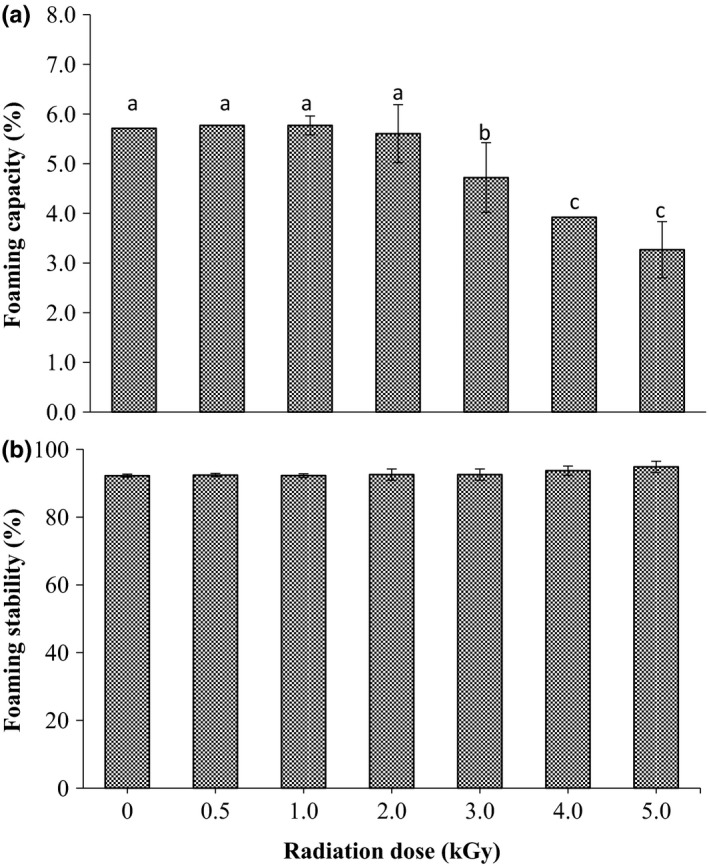
Effect of gamma irradiation on foaming capacity (a) and foaming stability (b) of sorghum. Values are means (±*SD*) of triplicate samples. Values followed by the same letter are not significantly different (*p* < 0.05) as assessed by LSD

The effect of gamma irradiation processing on the foaming properties is described in Figure 6a,b. The foaming capacity (FC) of the sorghum grains showed no significant (*p* < 0.05) change when they treated at the dose up to 2.0 kGy (Figure 6a). Increasing the radiation dose over 2.0 kGy significantly decreased the foaming capacity of the grains particularly at dose levels 4.0 and 5.0 kGy. While radiation treatment of the grains up to a dose of 5.0 kGy caused no significant changes in the foam stability (FS) of sorghum grains flour is presented in Figure 6b.

Our findings revealed that gamma radiation at doses up to 2.0 kGy cause no changes in FC of sorghum grains while higher doses were significantly reduced its FC. Similarly, Hassana et al. (2018) found that the FC of sesame seeds decreased after radiation treatment. In this study, decreasing of the foaming capacity of sorghum grain after exposure to gamma rays at doses more than 2.0 kGy might be due to the fact that change in the solubility and nature of protein occurred during ionizing radiation. According to Yasumatsu et al. (1972), gamma irradiation causes change in protein nature and this may lead to change in foaming properties. Regarding the foaming stability, our results showed that gamma irradiation did not affect the foam stability of sorghum grains. Several researchers also stated that gamma radiation caused no significant changes in foaming stability of soybean and velvet bean seeds (Pednekar et al., 2010; Bhat et al., 2008).

## 4 CONCLUSION

The obtained results concluded that gamma irradiation up to 5.0 kGy improved the storability properties of sorghum grains since it causes a severe reduction in the fungal growth and free fatty acids content of the grains. On the other hand, radiation of the sorghum grains up to 2 kGy enhanced its IVPD and protein solubility. Moreover, the emulsifying and foaming properties were not affected particularly at low doses. According to these results, therefore, low doses of gamma radiation could be applied as a safe alternative method for eliminating the fungal incidence in stored sorghum grains without degrading the protein quality and functionality.

## CONFLICT OF INTEREST

The authors declared no conflict of interest.
